# Clinical challenges in vaccine and monoclonal antibody development against ESCAPE pathogens: insights from EMA scientific advice procedures

**DOI:** 10.3389/fmed.2026.1931144

**Published:** 2026-10-09

**Authors:** Igor Stojkov, Linda Marchioro, Isabelle Bekeredjian-Ding, Benjamin Hofner

**Affiliations:** 1 Section Data Science and Methods, Paul-Ehrlich-Institut, Langen, Germany; 2 Division of Microbiology, Paul-Ehrlich-Institut, Langen, Germany; 3 Institute for Medical Microbiology and Hospital Hygiene, Philipps-University of Marburg, Marburg, Germany; 4 Department of Medical Informatics, Biometry and Epidemiology, Friedrich-Alexander-Universität Erlangen-Nürnberg, Erlangen, Germany

**Keywords:** clinical challenges, ESCAPE/ESKAPE pathogens, monoclonal anbitodies, scientific advice, vaccines

## Abstract

**Background:**

Combating ESCAPE pathogens (*E. faecium, S. aureus, C. difficile, A. baumannii, P. aeruginosa*, and *Enterobacteriaceae*) remains a major public health concern due to their treatment resistance and immune evasion. Vaccines and monoclonal antibodies (mAbs) are promising solutions, yet only one mAb is licensed and no vaccine has reached that stage. To identify recurring clinical challenges, we analyzed final advice letters (FALs) issued through the European Medicines Agency's (EMA) Scientific Advice procedure.

**Methods:**

We searched both the Paul-Ehrlich-Institut local database and the EMA database for FALs on vaccines and mAbs targeting ESCAPE pathogens. We included FALs that matched the pathogen and product criteria and addressed clinical development. We excluded those focused solely on quality, non-clinical, safety, or post-marketing aspects. From the included FALs, we extracted information across four main categories: primary endpoint, statistical analyses, study population, and study design. Regulatory responses were classified as fully, silently, partially, or not endorsed. We subsequently reviewed related clinical trial protocols to assess whether recommendations were implemented and to provide a narrative summary of changes to the development programs.

**Results:**

We included 14 FALs for each of the two medicine categories, related to six vaccine and 13 mAb trials. To address common challenges across the primary endpoints, regulators recommended selecting single, infection-related endpoints and emphasized precise clinical and laboratory case definitions to ensure attribution of infection to the target pathogen. They further advised on incorporating supportive evidence, such as subgroup analyses of cases or inflammation markers, direct microbiological or indirect measures (e.g., antibiotic use, hospital stay), or survival-related endpoints. Recommendations for study populations focused on refining the inclusion/exclusion criteria to identify those most likely to benefit. Regulators also advised on changes in the statistical analysis plan (mainly due to lower-than-expected incidence) and generally discouraged single pivotal trials.

**Conclusion:**

The development of vaccines and mAbs against ESCAPE pathogens is challenged by pathogen diversity, unpredictable infection rates, heterogeneous trial populations, and diagnostic uncertainty. In a field with no one-size-fits-all development pathway, early regulatory interaction and multidisciplinary disease-expert input are essential to reduce uncertainty and support robust, efficient clinical development programs.

## 1 Introduction

Antimicrobial resistance (AMR) is a major challenge to global health, impacting humans, animals, and the environment. Continuously recognized among the main public health threats (1, 2), 1.91 million deaths are expected to be attributable to AMR and 8.22 million associated with AMR by 2050, particularly affecting the elderly above the age of 70 (3). Even though antibiotics are essential to modern medicine, overuse and misuse have accelerated the development of AMR globally. Studies show that global antibiotic use increased by nearly 65% from 2000 to 2015 (4), and by an additional 16% from 2016 to 2023 (5), which was mainly due to increased consumption in low- and middle-income countries. Without effective intervention, antibiotic use is expected to further increase by 52% until 2030 (5). New reports from the World Health Organization (WHO) revealed that in 2023, antibiotic resistance was present in one out of every six laboratory-confirmed common bacterial infections (6). This escalating crisis highlights the need for a comprehensive, cross-sectorial approach to ensure that antibiotics remain effective for future generations (7).

In particular, ESKAPE pathogens (*E. faecium, S. aureus, K. pneumoniae A. baumannii, P. aeruginosa*, and *Enterobacter* species) have already demonstrated an ability to accumulate resistance features and “escape” available antibiotic treatments (8, 9). Following the proposed modification of the established ESKAPE acronym to include *C. difficile* (10, 11), the term ESCAPE is used to encompass this broader group (*E. faecium*, S*. aureus*, *C. difficile*, A*. baumannii*, *P. aeruginosa*, and *Enterobacteriaceae*—including *K. pneumoniae* and extraintestinal pathogenic *E. coli*-*ExPEC*). Their global impact with challenges in transmission, treatment, and prevention, along with their healthcare burden and emerging resistance trends, is reflected by high prioritization on the WHO Bacterial Priority Pathogens List (12) and the CDC Antibiotic Resistance Threats Report (13). Despite their structural heterogeneity, encompassing both Gram-positive and Gram-negative bacteria, ESCAPE pathogens share similarities as they are largely opportunistic pathogens and mostly affect immunocompromised and elderly individuals. Their wide-ranging clinical manifestations, including respiratory, urinary, gastrointestinal, and bloodstream infections, are associated with high morbidity and mortality, representing a critical challenge for healthcare systems.

In addition to the multiple, complementary actions for AMR prevention and optimized usage of available treatments (14–16), a crucial segment of AMR strategies lies in the sustainable investment into and development of novel treatment options, diagnostics, and surveillance platforms (17, 18). However, the number of antibacterials under development decreased from 97 in 2023 to 90 in 2025 (19), with only 15 considered innovative, and just five effective against WHO-defined critical drug-resistant bacteria. Vaccines, on the other hand, are known to be effective in preventing infectious diseases and reducing the need for antimicrobial drugs, and, thus, they have a great potential in helping to combat AMR (20, 21). Unlike antibiotics, vaccines are pathogen-specific, spare the microbiota and enhance herd immunity. These features reduce the risk of resistance development, and offer long-term cost savings. A recent WHO report highlights the potential of 44 vaccines to significantly reduce the AMR burden against 22 pathogens by preventing up to 408 thousand deaths annually, while concomitantly reducing antimicrobial use (22). Emerging alternatives to prevent or treat bacterial diseases in addition include monoclonal antibodies (mAbs), which target bacterial virulence factors rather than conserved surface proteins, thereby also reducing the selective pressure leading to resistance. While high development costs and limited coverage have posed significant challenges, recent advances, including companion diagnostics and multivalent antibody strategies, are improving their specificity and effectiveness (23). Additional promising strategies involve the use of bacteriophages, antimicrobial peptides (alone or in combination with antibiotics), therapeutic coinfection leveraging quorum sensing mechanisms and probiotic strategies etc (24).

In the last decades, numerous efforts have been made to develop vaccines, mAbs and alternative therapies to treat and prevent ESCAPE infections (24, 25). Still, only one of these development programs was successful as bezlotoxumab (commercial name Zinplava, by Merck, Sharp & Dome) was licensed for recurrent *C. difficile* infections (26). Given the low rates of success of the corresponding pivotal clinical trials, it is legitimate to wonder whether repeated or systematic bottlenecks in the design of such trials and development programs can be identified. We tackled this question by analyzing Scientific Advice (ScA) procedures, which allow medicine developers to proactively engage with experts from regulatory bodies responsible for licensure of medicines, such as the European Medicines Agency (EMA). Here, applicants submit their development plans for feedback on complex regulatory and scientific issues. Preliminary responses are generated by two national agencies, further consolidated by the multidisciplinary Scientific Advice Working Party, and afterwards discussed and adopted by the Committee for Medicinal Products for Human Use (CHMP). The official ScA is communicated to the applicant in a final advice letter (FAL) (27). Although voluntary and not legally binding, this early engagement with regulators was shown to strengthen development strategies and increase marketing authorization success (28–31).

In this study, we reviewed FALs related to the clinical development of vaccines and mAbs against ESCAPE pathogens and assessed their implementation in corresponding clinical trials. Our aim was to identify, from a regulatory perspective, common development challenges and explore solutions to improve and accelerate future medicine development and access to these products.

## 2 Methods

We conducted a document review (also known as document analysis) of FALs to assess their structure and content, involving document identification, data extraction, and descriptive analysis, followed by a narrative summary of the findings. We searched the Paul-Ehrlich-Institut local database and the EMA database [Scientific Explorer (32)] for FALs regarding initial or follow-up ScA on vaccines and mAbs/mAb-related products targeting ESCAPE pathogens. We first screened for inclusion based on whether the pathogen and product type matched the proposed search criteria. We included products targeting the pathogens of interest, including monoclonal and polyclonal antibodies, but excluded antimicrobial peptides and immunomodulators. We also excluded bacterial strains with different pathogenesis and epidemiology (i.e., Shiga toxin-producing or enterotoxigenic *E. coli*). We further assessed and included all FALs that addressed questions regarding clinical development plans, without restriction to the year of submission. We excluded FALs that concerned only quality, non-clinical, safety, or post-marketing aspects. From the included FALs, we extracted information relevant to four main categories: “Primary endpoint”, “Statistical analyses”, “Study population”, and “Study design”. The four main categories were derived from two online workshops on vaccine (2021) (33) and mAb (2022) (34) development, involving 21 and 24 speakers and moderators, respectively, fostering cross-pathogen learning across preclinical, translational, and clinical challenges. These four categories we subsequently divided into 21 subcategories, which were used in this document review. The data analysis included FALs issued up to June 2025. FALs from the Paul-Ehrlich-Institut database were identified within the bacterial vaccine and mAb ScA procedure folders, while FALs from the EMA database were retrieved using the EMA Scientific Explorer (search code provided in the Supplementary Information). We initially screened documents from both sources separately, after which we grouped potentially eligible FALs from both sources in a single Excel spreadsheet. We identified duplicates based on common characteristics (e.g., product identifier, bacterial pathogen, EMA case number), followed by final selection according to the predefined eligibility criteria. For the included FALs, we extracted product- and FAL-related information, as well as information across the 21 subcategories. Furthermore, we assessed and categorized the CHMP answers to the related subcategories as “Fully endorsed”, “Silently endorsed”, “Partially endorsed”, and “Not endorsed” (anonymized examples are provided in Supplementary Table S2). Two researchers (IS, HK, or LM) jointly conducted the screening, while the data extraction and CHMP answer categorization were performed independently. Disagreements were resolved during regular meetings throughout the data-extraction process, with consultation of a third researcher (LM, IBD, or BH) when necessary. Where multiple FALs related to the same product and trial (e.g., follow-up FALs), information within each category was counted only once unless the follow-up FAL introduced a new or modified proposal, in which case the change was recorded. We used frequencies and proportions to descriptively summarize findings at an aggregate level, providing an overview of observed patterns.

In a subsequent step, we searched the Paul-Ehrlich-Institut local database for the FAL-related clinical trial protocols and amendments, and stratified the results according to whether the trial had been initiated or not. For all trials that were initiated and contained partially or non-endorsed CHMP recommendations, the identified protocols and amendments were reviewed to determine if the CHMP recommendations on the clinical trial design had been implemented. These discussion points and the corresponding protocols and amendments were compiled in an Excel spreadsheet and ordered by document date. The search and initial assessment were performed by one researcher (IS), with the summary information subsequently reviewed and discussed with the other researchers (BH, LM, and IBD) during manuscript preparation.

To maintain the confidentiality of the FALs and clinical protocols, our results do not disclose applicant's identity, product name, or specific indication. Furthermore, we use the term “applicant” to refer broadly to the medicine developer, sponsor, or any other party submitting a request to the EMA, or making changes in the trial protocols.

## 3 Results

Following the initial screening for pathogen and product relevance in each database, we identified 37 vaccine FALs and 25 mAb FALs. After removing duplicates and excluding those without a clinical focus, 14 FALs fulfilled the inclusion criteria for both vaccines and mAbs (Figure 1).

**Figure 1:**
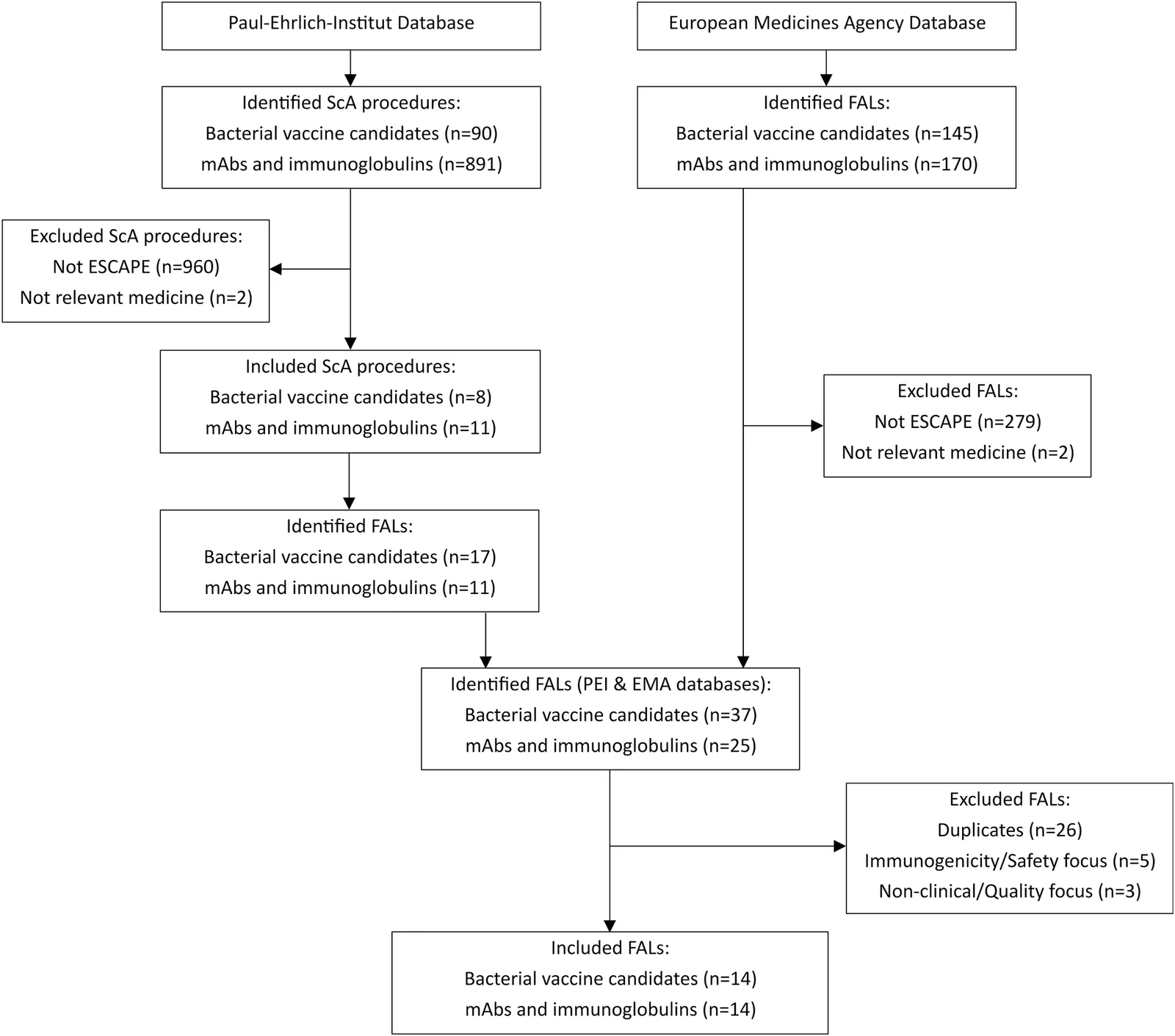
Flow chart describing the screening process of the final advice letters. EMA, European medicines agency; ESCAPE pathogens, (*E. faecium, S. aureus, C. difficile, A. baumannii, P. aeruginosa, and Enterobacteriaceae—*including *K. pneumoniae* and extraintestinal pathogenic *E. coli*-ExPEC); FAL, final advice letter; mAb, monoclonal antibodies; PEI, Paul-Ehrlich-institut; ScA, scientific advice procedure.

### 3.1 Description of FALs, corresponding clinical trials, and CHMP feedback

#### 3.1.1 Vaccines

We identified 14 relevant FALs, associated with five products and four pathogens (*C. difficile, ExPEC, P. aeruginosa, S. aureus*), as presented in Table 1. These 14 FALs, including three initial and eleven follow-ups, were submitted between 2006 and 2024 (Supplementary Figure S1). They addressed a total of 67 questions, 44 of which were related to the clinical development, from which we mapped 116 discussion points. This number surpasses the total number of questions, as many were multifaceted and included multiple areas of discussion. The 116 discussion points were related to six vaccine trials, including two Phase 2 and two Phase 3 trials that were initiated, and two Phase 3 trials that were not initiated (Table 1).

**Table 1:** Summary characteristics of the included final advice letters and clinical trials.

	Vaccines	mAbs
Final advice letter	*n* = 14	*n* = 14
Primary	3	9
Follow-up	11	5
Assessed pathogen	*n* = 14	*n* = 14
*C. difficile*	4	2
*ExPEC*	5	/
*P. aeruginosa*	3	3
*S. aureus*	2	9
Assessed products	*n* = 5	*n* = 9
Prevention	5	6
Treatment	/	3
Assessed trials	*n* = 6	*n* = 13
Phase III	4	10
Phase II^a^	2	3
Initiated		
Phase III	2	4
Phase II	2	2
Not initiated		
Phase III	2	6
Phase II	/	1

ExPEC, extraintestinal pathogenic *Escherichia coli*; mAb, monoclonal antibodies.

a Including Phase IIb or Phase II/III trials.

Across the products, discussion points where CHMP disagreed with the company proposal (i.e., partially endorsement/no endorsement) in more than three occasions (i.e., frequent disagreement points) were related to the use of a single pivotal trial (*n* = 10), interim analyses (*n* = 9), power calculations (*n* = 6), target vaccine efficacy (*n* = 6), effect estimation (*n* = 5), and proposed time frame (*n* = 4). Less frequent but potentially important points of disagreement (occurring once or twice) were related to the clinical definition of the endpoint, comorbidity eligibility criteria, stratification, and multiplicity control. Since almost all vaccine trials were initiated at some point, we observed a similar pattern in the discussion points in initiated trials in comparison to all trials (Figure 2 and Supplementary Table S3).

**Figure 2:**
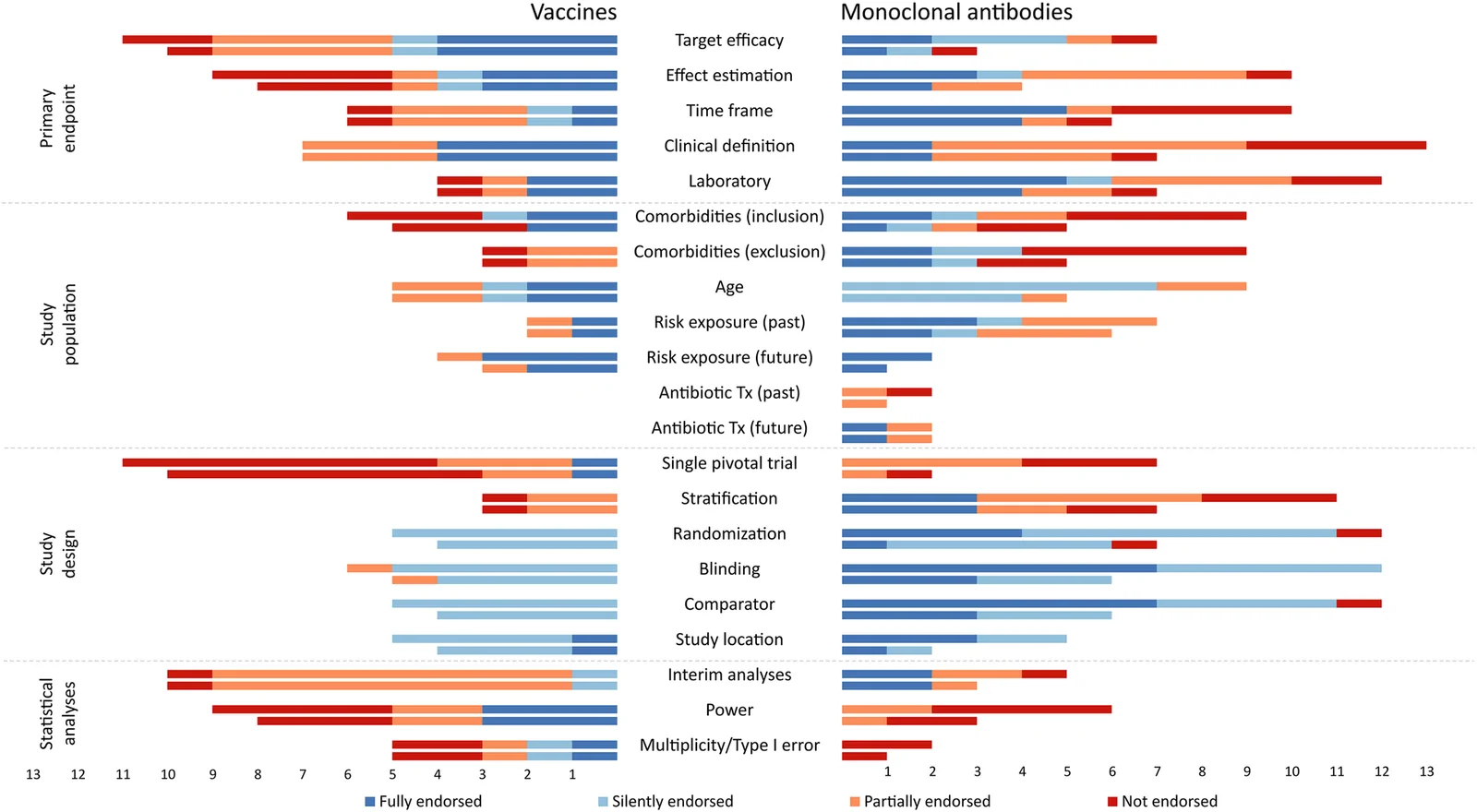
Butterfly diagram of the FAL discussion points and level of endorsement, among the four categories and 21 subcategories, stratified by discussion points across all clinical trials (top bar), and among initiated trials (bottom bar). FAL, final advice letter; Tx, treatment.

#### 3.1.2 Monoclonal antibodies

In the same manner, 14 FALs related to mAbs fulfilled the inclusion criteria. They were related to nine products (six preventive, three therapeutic), three pathogens (*C. difficile, P. aeruginosa, S. aureus*), and were submitted between 2007 and 2024 (see Table 1 and Supplementary Figure S1). These 14 FALs (nine initial and five follow-ups) addressed 104 questions, of which 71 were related to clinical development, resulting with 164 mapped discussion points. These discussion points were related to 13 trials, including two Phase 2 and four Phase 3 trials that were initiated, and one Phase 2 and six Phase 3 trials that were not initiated (Table 1).

Frequent points of disagreement included the clinical definition of the primary endpoint (*n* = 11), stratification at baseline and analyses (*n* = 8), and relying on a single pivotal trial (*n* = 7), along with power calculations (*n* = 6), and comorbidity eligibility criteria (*n* = 5-6). Additionally, many of these frequent disagreements were further related to the primary endpoint, i.e., effect estimation, time frame, and laboratory aspects (*n* = 5-6). Planned interim analyses and past risk exposure each raised disagreement in three cases. The points of disagreement were more frequent in the trials that were not initiated, highlighting the need for further research and planning (Figure 2 and Supplementary Table S3).

### 3.2 Detailed review of CHMP recommendations

#### 3.2.1 Vaccines—initiated trials

Within the “Primary endpoint” category, CHMP recommendations to revise the endpoints, emphasizing more precise case ascertainment, were raised across all four trials. For example, the CHMP suggested clearer guidance on case descriptions based on potential symptoms and how participants and medical practitioners should assess them, requested the use of standardized confirmatory or duplicate tests across laboratories, as well as independent endpoint adjudication. All these recommendations were implemented by the applicants.

In one Phase 3 trial, the CHMP advised to choose a single primary endpoint instead of introducing a hierarchical second primary endpoint. Although the applicant did not adopt this advice at the trial beginning, they implemented this change before the first interim analysis, which was considered acceptable by the CHMP as the applicant had not previously reviewed any efficacy data.

In another pivotal trial, the CHMP endorsed a time-to-event primary endpoint beyond infection. Examples of such endpoints include time to hospitalization, clinical deterioration, or mortality. However, it was noted that no previous study in a similar setting had shown an effect of the expected magnitude on this endpoint and highlighted the risks of relying on a single pivotal study, particularly if there is no supporting evidence of preventing the targeted infections. The CHMP also suggested using a full analysis set (i.e., all randomized participants) in the primary analysis, which the applicant adopted. Still, the interim results indicated a smaller benefit than expected, raising concerns over the sufficiency of evidence for a single pivotal trial. This triggered a discussion about a sample size adjustment. However, as it was not prespecified and no adequate plan for the control for type I error was provided, this led to further concerns on the evidentiary standards as well as trial integrity. Therefore, the CHMP recommended a second pivotal trial, emphasizing selection of participants most likely to benefit from the product (thus enhancing the potential to show a treatment benefit), and prioritizing infection-related endpoints, either direct microbiological endpoints, or more indirect measures (e.g., antibiotic use, hospital stay).

The CHMP also discussed the follow-up length in three trials, approving extended follow-up in two cases to assess lasting immunity or to accrue sufficient cases when justified, as long as there was adequate control of the overall type I error. In the third case, the CHMP recommended initiating a new trial rather than extending the current one for several years and enhancing subject retention, as proposed by the applicant, due to the low likelihood that the current trial population would be exposed to the pathogen.

In the “Study Population” category, the CHMP often suggested refinements in the inclusion/exclusion criteria to further specify the trial population, such as: not to enroll patients with ongoing infections or those with specific antibiotic therapy related to the vaccine pathogen, to clarify the timeframe for the exclusion criteria, and to ensure the criteria for suspected cases at baseline are clear, scientifically robust, and standardized. All these recommendations were incorporated into the clinical trial protocols, including the removal of a proposed scoring system at initial enrolment, following CHMP concerns related to the use of a specific cut-off point.

Within the “Study design” category, the CHMP provided guidance on stratification for two Phase 3 trials. In one case, the recommendation from the CHMP to include the study sites as strata in the randomization was implemented in the protocol whereas the analysis was adjusted for country due to the low number of participants per site.

In another trial, based on the suggestions from the CHMP, the applicant provided details on the age stratification and baseline risk factors, and excluded stratification according to study center as there was no clear rationale.

Furthermore, the possibility of approval based on a single pivotal trial raised serious concerns in three of the four trials, especially when the proposed changes could compromise the robustness and reliability of the findings. This was the case when it was planned to demonstrate vaccine efficacy with substantially fewer cases than previously anticipated, to increase the sample size with unjustified inclusion criteria or without appropriate type I error control, to make design changes towards an open-label, non-comparative trial upon positive interim results, or when Phase 2 trials were lacking as supporting evidence.

Within the “Statistical analyses” category, a recurring issue identified across all initiated trials was a lower-than-expected incidence rate. Consequently, the applicants proposed increasing participant enrolment, reducing the required number of primary cases, or the implementation of additional interim analyses to allow for earlier success or futility assessment. These modifications were in principle endorsed by regulators, provided that the success criterion was maintained. The regulators also emphasized the importance of appropriately justifying type I error control, preserving trial integrity, and minimizing the introduction of bias.

#### 3.2.2 Monoclonal antibodies—initiated trials

Since this analysis focuses on areas of disagreement, one of the initiated Phase 3 trials was excluded because the CHMP agreed with the proposed plan. As a result, we reviewed the remaining five initiated trials (two Phase 2 and three Phase 3), all of which included CHMP concerns within the “Primary endpoint” category, particularly regarding diagnostic accuracy of case ascertainment and assurance that the infection is primarily or wholly caused by the pathogen of interest.

For example, in one Phase 2 trial, the CHMP endorsed the rate of cases as primary endpoint but noted concerns regarding the diagnosis and supported exploring an all-cause mortality endpoint for this earlier phase. Although this is less specific, it avoids uncertainties in diagnosis and etiology. The applicant addressed this by including multiple exploratory outcomes related to all-cause mortality, different infection subgroups, comparison of colonization strains at screening and strains present at diagnosis etc.

In another Phase 2 trial, initial consultations with the CHMP were generally positive. Post-interim analysis, consultations mainly focused on the potential use as of the study as a pivotal trial. This raised several issues. While this change was found to be acceptable, the CHMP recommended further protocol specifications such as clarifying whether patients with mixed cultures containing the pathogen of interest would still be defined as cases in the primary endpoint. The assessment of all infections regardless of the pathogen, as well as all-cause mortality outcomes were advised as secondary rather than exploratory endpoints. Changes to the statistical analysis plan and sample size recalculations were also requested.

Other suggestions from the CHMP across the Phase 2 trials, included comparing clinical signs of severity or inflammation in the active and placebo groups, and clarifying the imputation strategy (i.e., counted as cases or failures) for participants who discontinued the trial before the primary outcome was observed or who received antibiotics for the target pathogen in other body systems. All of this was addressed by incorporating additional sensitivity analyses in the protocols.

To address the CHMP suggestions for the Phase 3 trials, one trial replaced the initially proposed two primary endpoints with a single primary endpoint. In another trial, the CHMP highlighted potential uncertainty about the etiology of infection in the primary endpoint definition, which remained despite the secondary endpoint for specific pathogen-related infection. In the third Phase 3 trial, it was recommended to assess the duration of protection in the extended twelve-month phase, which was implemented in the protocol.

Within the “Study population” category, the CHMP recommendations were related to the three Phase 3 trials.

In one trial, the applicant clarified the standard-of-care therapy including the dosing of antibiotics, and revised the inclusion criteria in order to enrol patients with higher chances for the outcome of interest.

In the second trial, the CHMP noted that the upper age limit for inclusion might be unjustified and could restrict marketing authorization, but the criterion was retained in the protocol.

For the third trial, recommendations focused on clarifying prior antibiotic usage as an exclusion criteria and standardizing diagnostic procedures across sites, including the use of tests with similar sensitivity and specificity and evaluating their performance through secondary or exploratory analyses. The applicant implemented further changes and clarifications regarding these two points. However, the CHMP suggestion to exclude patients with positive antigen tests for specific coinfections, intended to strengthen evidence that the infection was attributable to the target pathogen, was not followed. The applicant stated that these coinfections would be treated accordingly and that, due to the distinct mode of action of the evaluated mAb, there would be no interference with an adjunctive antibiotic therapy.

For the “Study design” category, the CHMP proposed changes in three of the five trials. These were related to the stratification rules and their usage in the models, which were considered and partly resolved in two cases, whereas in one case the “likeliness of disease progression at baseline” was kept in the trial protocol despite the suggestion that it may be subjective and of unclear value.

In terms of the “Statistical analyses” category, CHMP recommendations were related to one Phase 2 trial and two Phase 3 trials.

Main challenges for the Phase 2 trial included the impact of dose selection on the statistical analysis, e.g., with regard to type I error control, as well as the newly proposed sample size re-assessment and interim analyses timing. These issues were resolved in the trial protocol. Regulators also advised that if the trial is to be considered pivotal, the two-sided significance level should be adjusted from 0.1 to the conventional standard of 0.05. This was considered particularly critical if the trial was planned to be used as a single pivotal trial, which was generally not supported.

Similarly, in one of the Phase 3 trials, the main issue was the use of a confidence interval based on a two-sided significance level of 0.1 for a potential single pivotal trial. The applicant addressed this in the protocol by adopting a one-sided significance level of 0.025.

In the other Phase 3 trial, the concern regarding the analytical approach with two primary endpoints was resolved by defining a single primary endpoint.

#### 3.2.3 Vaccines and monoclonal antibodies–not initiated trials

Among the planned vaccine trials (two Phase 3 trials), one applicant asked whether approval could be granted based on positive results from a planned Phase 3 trial conducted after the completion of an ongoing Phase 2 trial, which the CHMP considered likely.

In the other Phase 3 trial, the applicant proposed raising the significance level for the primary endpoint to 0.1 and using comparisons with registry data, as supportive evidence of efficacy. These changes were suggested in response to lower-than-expected outcome rates, likely due to improved treatment and preventive measures since the trial planning. Regulators did not support these changes, and noted that increasing the significance level following access to unblinded data is statistically unacceptable, and that comparisons with registry data are prone to bias and require clear definition of controls. Regulators also raised concerns regarding the clinical relevance of the primary outcome, and advised on the secondary efficacy endpoints.

Among the planned mAb trials (one Phase 2 and six Phase 3 trials), the CHMP identified multiple study design issues in one of the Phase 3 trials that were not initiated. These issues were related to an unclear primary endpoint, no specified diagnostic test for pathogen detection, unclear duration of prior antibiotic treatment, missing product-related exclusion criteria, interim analyses without control of the overall type I error, and inadequate randomization planning.

For the other six trials, advice focused on refining the definition and timing of the primary endpoint, detailed sample size estimates, avoiding mortality as primary endpoint (as this would require very large sample size), or use of external control for efficacy, standardizing concomitant antibiotic use and diagnostics across sites, as well as aligning the indication, supporting evidence, and primary objectives of the analysis. Regulators also discussed narrowing inclusion criteria to patients with known or strongly suspected primary infection foci/relevant prognostic factors, to reduce dilution of treatment effect from comorbidities and colonization status. They discouraged use of multiple stratification factors in the randomization, as this would not be an effective way to address heterogeneity, and recommended focusing on the most important prognostic factors. Overall, multiple design issues were noted across these trials, and relying on a single pivotal trial was generally not supported.

## 4 Discussion

This document review outlined the key clinical challenges related to showing efficacy of vaccines and mAbs against ESCAPE pathogens, as discussed in EMA ScA procedures. Analysis of the FALs revealed frequent alignment between regulators and applicants on blinding, study location, randomization (except when randomization procedures were unclear or changes of the allocation ratio were planned), and choice of comparator arm (with the exception of the use of external control cohorts).

By contrast, subcategories within the “Primary endpoint” had multiple, common issues across products. According to the EMA vaccine guidelines, definition of the primary endpoint in the absence of an established correlate of protection “usually comprise[s] clinical signs and/or symptoms typical of the infectious disease together with laboratory confirmation of the aetiology” (35). The case definition should, thus, reflect the clinical features and symptoms of the disease in line with the proposed indication, especially in infections with varying severity or disease manifestations. Similarly, for prophylactic and therapeutic mAb products, the guideline for treatments of bacterial infections (36) suggests to use clinical and/or microbiological outcomes as primary endpoints in accordance to the type of infection, with examples in specific indications and in general. This is aligned with the WHO mAb guideline (37), which emphasizes that endpoints should be specific to the product and pathogen, clinically meaningful, patient-centered, preferably laboratory-confirmed, and should reliably demonstrate benefit compared to an appropriate comparator. In line with this guidance, the suggestions from the regulators primarily were to focus on a single, well-defined primary endpoint, with a clear clinical definition of the expected signs and symptoms. Preference was given to pathogen-related primary endpoints that align with the products’ mechanism of action, rather than survival-related outcomes. The attempt to specify outcomes to align with the mechanism of action avoids uncertainty due to imprecise diagnosis, which bears the risk of trial failure due to cases that cannot be cured or prevented by the medicinal products. This approach is especially important in view of the heterogeneity of the study population, which increases the risk of obtaining inconclusive study results through diverse underlying comorbidities, varying standards of care and varying diagnostics across sites, and differing immune status of patients (e.g., immunosuppressed or older participants).

In addition, the diagnostic accuracy of biomarkers at baseline and to assess endpoints was repeatedly discussed, particularly for mAb products. This was crucial for studies intended as pivotal trials, as it was unclear whether a mixed culture, containing both the pathogen of interest and other bacteria, is acceptable to fulfil the microbiological criterion of the primary endpoint. This reflects the inherent diagnostic challenge of distinguishing invasive infection and secondary infections from colonization of mucosa. According to the EMA guidelines, “protocols should list the pathogens that may be considered causative in the type of infection under study” (36), and central laboratory data should be used for outcome analyses referring to the respective pathogens and their *in-vitro* susceptibility. This can range from monitoring of bacterial growth in conjunction with pathogen identification and *in vitro* susceptibility testing to quantifiable pathogen-specific molecular detection methods. Similarly, for vaccines, case confirmation should use validated, predefined lab methods with high accuracy, and may include tests for coinfections where relevant (35). Still, the methods to determine whether an infection is caused solely by the target pathogen, another organism, or coinfections, are not and cannot be fully specified in the guidelines, as these depend on pathogen characteristics, available diagnostic tools, local epidemiology and clinical presentation of the patients. Prior collaborative input from microbiologists, infectious disease specialists, and reference laboratories is often necessary to accurately classify infections in clinical trials. Expert involvement in steering committees or endpoint adjudication committees prior to trial initiation can be particularly valuable for the trial integrity and unbiased endpoint assessment. Furthermore, standardized testing practices across clinical sites were also requested for baseline assessment since different diagnostic approaches will likely add to study complexity. However, diagnosis and treatment of an infection is time critical and usually performed on site. This has favored the use of point-of-care diagnostics in clinical trials to ensure a timely treatment and standardized on-site assessment, while centralized laboratories often only retrospectively confirm or reject the initial diagnosis in order to achieve comparability across study sites. Moreover, while randomization is used to mitigate possible study arm imbalances, false positive cases can negatively impact the balance and subsequently the efficacy results.

In terms of the “Study population”, care should be taken to define inclusion/exclusion criteria to select study participants who are most likely to benefit from the products. For example, applicants were advised to avoid vaccination of participants with ongoing infections, sepsis, or those receiving antibiotic therapy related to the vaccine pathogen. In therapeutic mAb trials, patients who have recently received effective antibiotic treatment for the target pathogen prior to randomization should generally be excluded, unless previous therapy was documented as ineffective. Additionally, the standard of care dosing regimen should be standardized across all participants.

With regard to the “Study design”, discussion points on baseline covariates addressed their use in both the design stage and analysis. Adjustment for baseline covariates (e.g., age, country, study site, risk factors) can be incorporated via stratified randomization and/or stratified or covariate-adjusted analyses. Adjustment is recommended when there is a strong or moderate association with the primary outcome, as it improves analytical efficiency and mitigates bias caused by baseline covariate imbalances that may occur by chance. The selection of covariates should be pre-specified and kept to a minimum. If stratified randomization is used, the stratification factors should also be included in the primary analysis (unless they were included only for administrative reasons), with any differences well justified. Adjusted analyses should be also accompanied by subgroup and sensitivity analyses, in order to assess the model assumptions. Any discrepancies in effect estimates should be discussed (38, 39).

In cases where only one pivotal study is planned for a marketing authorization application, the study must demonstrate good internal and external validity (minimization of bias and generalizability of findings), clear clinical relevance (substantial treatment benefit), and strong statistical significance, often requiring a significance level well below the standard 0.05 with precise effect size estimates. Moreover, high-quality data and consistent findings across all key endpoints, subgroups, and study centers are necessary. And while a single pivotal Phase III trial with statistically robust and clinically meaningful results may suffice for a marketing authorization application in principle, its acceptability will always depend on a case-by-case assessment of the data at the time of submission, following the EMA guidance on single-trial applications (40). Planning for more than one study is generally advisable, particularly in cases of unknown or novel mechanisms of action, limited or inconclusive early-phase data, a history of failed trials in the therapeutic area, or when efficacy and safety need to be established across multiple subpopulations or comparators. In addition, the study data will later be subject to evaluation by health technology agencies, payers and recommending medical associations.

In terms of the “Statistical analysis” category, study design adaptations based on interim results, such as sample size re-estimation, adaptations to trial population, or sequential analyses for early stopping, were advised in line with established (38, 41) and developing (42) guidelines. Methods need to be pre-specified to ensure that the results from different stages of the trial can be justifiably combined, bias is avoided or at least reduced, and the type I error of the study is protected. In general, the success criterion (for example, vaccine efficacy greater than 20%) and the group sequential spending function should be pre-specified and preserved throughout the trial. Furthermore, there is a risk of bias from unblinding, which could lead to informative dropout or decisions influenced by early data, and ultimately affect the validity of the results. To avoid this, robust measures should be in place to ensure that efficacy information is not disclosed or used to change the trial design. Additionally, since duration of protection is a relevant aspect for marketing authorization, it is generally advised that, even following a positive confirmatory analysis (43), the study continues with blinded follow-up of all participants to ensure that long-term efficacy and safety can be properly assessed. It is important to distinguish between planned adaptations within adaptive trial designs and unplanned changes introduced outside the protocol. Changes to the study design that were not pre-specified are usually only permissible if these are based on external or blinded information. If they are based on trial data (e.g., when increasing the sample size based on interim results), they are usually not supported as they pose substantial risks to the validity and robustness of the overall results and can inflate the type I error. It is therefore strongly recommended that unplanned data-driven changes are avoided.

Several limitations should be considered when interpreting our study findings. The systematic categorization into 21 subcategories, although established in agreement with expert opinions, may include some overlap or interdependency between topics. To minimize bias, we used standardized extraction protocols, independent reviews, and consensus discussions. Nevertheless, we may have missed some relevant discussion points, and discussion points may have overlapped across follow-up FALs related to the same trial. In addition, the assessment of regulatory endorsement can involve subjective judgement. Therefore, the reported frequencies and proportions should be interpreted as descriptive context rather than formal statistical estimates, and should be interpreted accordingly. Moreover, as the review of the subsequent implementation of CHMP recommendations in clinical trial protocols was primarily conducted by one researcher, potential information bias cannot be excluded, despite the result discussions among co-authors. Additionally, FALs are primarily intended to support marketing authorization and are independent of clinical trial registration and amendment processes. Therefore, FAL recommendations may not always align with subsequent protocol amendments, and any improvements in trial conduct or outcomes cannot be attributed solely to the incorporation of the FAL recommendations into the protocol. Confidentiality requirements limited our ability to report detailed information on individual products or pathogens, as well as more specifics on their product development or related changes. We also did not investigate the company-specific reasons for not implementing regulatory recommendations in the trial protocols, since this was beyond the study aim, and would require a separate assessment with consensual input from all applicants, conducted in a trustworthy and informative manner. Furthermore, our analysis was limited to products for which EMA ScA was requested, and did not incorporate perspectives from regulatory authorities outside the EMA framework. This selection, of course, reflects a regional focus on relevant nosocomial diseases. In line with this, for example, we did not find any ScA procedures for *K. pneumoniae*, which is also a current target for vaccine development. It should also be noted that ScA is voluntary, and therefore the included cases may not represent all vaccine and mAb development activities in this field. Changes in regulatory guidance and clinical practice over the study period should also be considered when interpreting the findings.

## 5 Conclusion

As shown in this review, along with insights from previous expert workshops (33, 34), and published literature (21, 44), developing vaccines and mAbs against ESCAPE or hospital-acquired bacterial infections is highly complex, both due to pathogen-specific characteristics (e.g., antigen diversity) and to clinical trial aspects (e.g., unpredictable infection rates, participant heterogeneity). Diagnostic limitations further hinder the accurate selection of trial participants and complicate distinguishing between infection, colonization, and co-infections. Taken together, these obstacles highlight the critical need for rapid diagnostics, standardized immune measurements, robust multi-center and multi-regional trial networks, and precisely defined clinical outcomes. While the EMA guidance documents provide valuable general recommendations and a balance between scientific rigor and flexibility, defining infection- and product-specific characteristic within the study design still largely relies on expert input and prior knowledge, especially from Phase 2 trials. Since there are many uncertainties and no one-size-fits all concept, early interaction with regulators and other developers is particularly valuable to avoid ineffective trials. Our document review can help to make future developers aware of the obstacles to more precisely plan late-stage clinical trials and prepare for regulatory interactions, ultimately supporting the accelerated development of life-saving therapies.

## Data Availability

The data analyzed in this study is subject to the following licenses/restrictions: The data supporting this study are not publicly available as they contain confidential and proprietary information from medicine developers. While main aspects of ScA procedures are provided in the European Medicines Agency’s public assessment report when a product is licensed, only one product in the current study has been licensed, and therefore more detailed supporting materials remain unavailable. Access to data is limited to authorized representatives or affiliates of the European Medicines Agency and national medicines regulatory authorities.
