# Supplementary material for: Clinical challenges in vaccine and monoclonal antibody development against ESCAPE pathogens: insights from EMA scientific advice procedures
Source: Front Med (Lausanne). 2026 Oct 9;13:1931144. doi: 10.3389/fmed.2026.1931144 (PMC13692650; doi:10.3389/fmed.2026.1931144)
Supplement: Supplementary file 1 [file Supplementaryfile1.pdf]

Supplementary information

EMA Scientific Explorer search code - Vaccines:
(Document text contains 'faecium' OR Document text contains 'aureus' OR Document text contains 'pneumoniae' OR Document text contains 'difficile' OR Document text contains 'baumannii' OR Document text contains 'aeruginosa' OR Document text contains 'enterobacter' OR Document text contains 'enterobacteriaceae' OR Document text contains 'coli') [AND] (Substance/product class equals 'Vaccines').

EMA Scientific Explorer search code - Monoclonal antibodies:
(Document text contains 'faecium' OR Document text contains 'aureus' OR Document text contains 'pneumoniae' OR Document text contains 'difficile' OR Document text contains 'baumannii' OR Document text contains 'aeruginosa' OR Document text contains 'enterobacter' OR Document text contains 'enterobacteriaceae' OR Document text contains 'coli') [AND] (Substance/product class equals 'Immunoglobulins' OR Substance/product class equals 'Antibodies' OR Substance/product class equals 'Immunoconjugates' OR Substance/product class equals 'Immunoglobulin constant regions' OR Substance/product class equals 'Immunoglobulin Fc fragments' OR Substance/product class equals 'Immunoglobulin fragments' OR Substance/product class equals 'Fab fragments' OR Substance/product class equals 'Immunoglobulin Fv fragments' OR Substance/product class equals 'Immunoglobulin fusion proteins').

Supplementary Figure 1. Years in which the final advice letters included in the review were issued

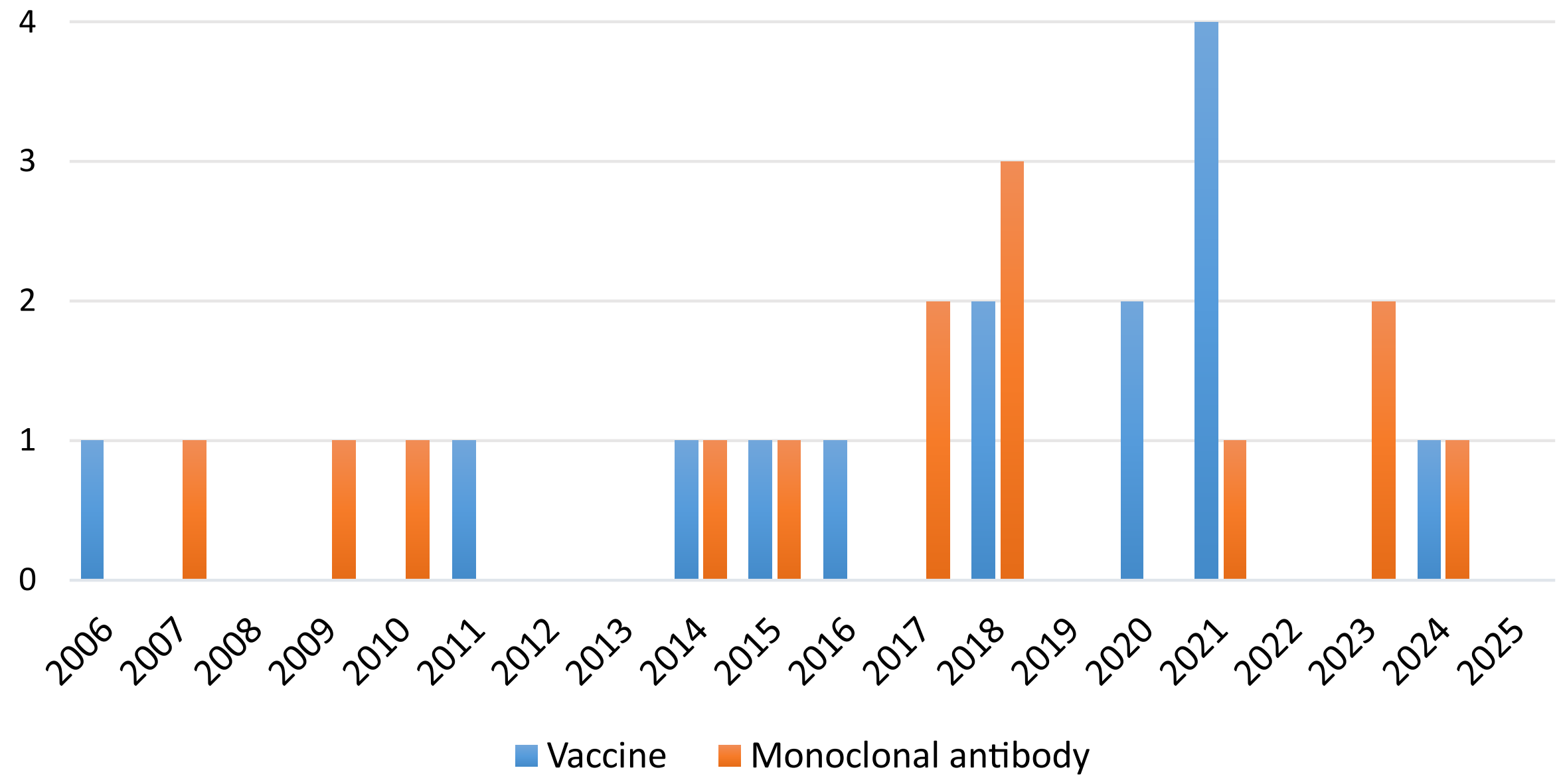

Supplementary Table 1. Overview of extraction criteria for the 21 subcategories

**Primary endpoint category**	
Clinical definition	Clinical symptoms and clinical case definitions
Effect estimation	Estimand (i.e., trial objective/main treatment effect) or estimator (i.e., the method of analysis to compute an estimate)
Laboratory	Microbiological assessment procedures, laboratory tests, and mechanisms for case ascertainment
Target efficacy	Related to mAb/vaccine effect size
Time frame	Time frame of the primary endpoint or the time of measurement
**Statistical analyses category**	
Interim analyses	Interim analysis approach
Multiplicity/Type I error	Multiplicity or type I error control
Power	Power/sample size estimation
**Study population category**	
Age	Study population age
Antibiotic treatment (past)	Trial inclusion criteria based on past antibiotic treatment
Antibiotic treatment (future)	Trial inclusion criteria based on planned or expected antibiotic treatment
Comorbidities (inclusion)	Trial inclusion criteria based on past or recent comorbidity/microbiological diagnosis
Comorbidities (exclusion)	Trial exclusion criteria based on past or recent comorbidity/microbiological diagnosis
Risk exposure (past)	Trial inclusion criteria based on past or recent events (e.g., ICU or hospital admission, mechanical ventilation)
Risk exposure (future)	Trial inclusion criteria based on planned or expected events (e.g., planned hospitalization or surgery)
**Study design category**	
Blinding	Blinding approach
Comparator	Trial comparator/control group
Randomization	Randomization procedure and allocation ratio
Single pivotal trial	Use of a single confirmatory pivotal trial for marketing approval
Stratification	Baseline stratification or use of stratified analyses
Study location	Trial setting (e.g., multicentric, multiregional)

ICU - Intensive care unit; mAb - monoclonal antibody

Supplementary Table 2. Examples of the categorization of CHMP responses

Endorsed	"This proposal for a primary analysis, which is therefore confined to those who received all xxx doses, would be acceptable in a vaccine efficacy trial."<br>"For the purposes of a Phase 2 study the patient selection criteria are acceptable, including the allowance for prior anti-xxx therapy within the specified limits."
Silently endorsed	Silently endorsed referred to aspects of a proposed study design that were not explicitly addressed in the CHMP response but were included in the applicant's question. For example, if an applicant's question referred to a randomization method or study location among other aspects, and the CHMP did not explicitly comment on these aspects but supported the overall study design, they were considered silently endorsed.
Partially endorsed	"The type and duration of SoC antibiotic therapy is acceptable. Doses of antibiotic should be standardized."<br>"In principle, the proposed strategy for sample size re-assessment is acceptable but the exact timing of each interim analysis should be pre-specified in the protocol."
Not endorsed	"The CHMP does not agree with the inclusion of participants without evidence of xxx pathogen in the blood or in any other sterile site in the first composite of the primary endpoint. "<br>"The applicant plans two interim analyses which are currently not considered in the sample size calculation and no multiplicity control for the type I error is foreseen, which is not endorsed"

CHMP - Committee for Medicinal Products for Human Use; SoC - Standard of Care

Supplementary Table 3. FAL discussion points and level of endorsement, among the four categories and 21 subcategories. Numbers above the diagonal line refer to all clinical trials while numbers below the diagonal refer to initiated trials only.

Vaccines					Monoclonal antibodies			
Not endorsed	Partially endorsed	Silently endorsed	Fully endorsed		Fully endorsed	Silently endorsed	Partially endorsed	Not endorsed
2 / 1	4 / 4	1 / 1	4 / 4	Target efficacy	2 / 1	3 / 1	1 / 0	1 / 1
4 / 3	1 / 1	1 / 1	3 / 3	Effect estimation	3 / 2	1 / 0	5 / 2	1 / 0
1 / 1	3 / 3	1 / 1	1 / 1	Time frame	5 / 4	0 / 0	1 / 1	4 / 1
0 / 0	3 / 3	0 / 0	4 / 4	Clinical definition	2 / 2	0 / 0	7 / 4	4 / 1
1 / 1	1 / 1	0 / 0	2 / 2	Laboratory	5 / 4	1 / 0	4 / 2	2 / 1
3 / 3	0 / 0	1 / 0	2 / 2	Comorbidities (inclusion)	2 / 1	1 / 1	2 / 1	4 / 2
1 / 1	2 / 2	0 / 0	0 / 0	Comorbidities (exclusion)	2 / 2	2 / 1	0 / 0	5 / 2
0 / 0	2 / 2	1 / 1	2 / 2	Age	0 / 0	7 / 4	2 / 1	0 / 0
0 / 0	1 / 1	0 / 0	1 / 1	Risk exposure (past)	3 / 2	1 / 1	3 / 3	0 / 0
0 / 0	1 / 1	0 / 0	3 / 2	Risk exposure (future)	2 / 1	0 / 0	0 / 0	0 / 0
0 / 0	0 / 0	0 / 0	0 / 0	Antibiotic Tx (past)	0 / 0	0 / 0	1 / 1	1 / 0
0 / 0	0 / 0	0 / 0	0 / 0	Antibiotic Tx (future)	1 / 1	0 / 0	1 / 1	0 / 0
7 / 7	3 / 2	0 / 0	1 / 1	Single pivotal trial	0 / 0	0 / 0	4 / 1	3 / 1
1 / 1	2 / 2	0 / 0	0 / 0	Stratification	3 / 3	0 / 0	5 / 2	3 / 2
0 / 0	0 / 0	5 / 4	0 / 0	Randomization	4 / 1	7 / 5	0 / 0	1 / 1
0 / 0	1 / 1	5 / 4	0 / 0	Blinding	7 / 3	5 / 3	0 / 0	0 / 0
0 / 0	0 / 0	5 / 4	0 / 0	Comparator	7 / 3	4 / 3	0 / 0	1 / 0
0 / 0	0 / 0	4 / 3	1 / 1	Study location	3 / 1	2 / 1	0 / 0	0 / 0
1 / 1	8 / 8	1 / 1	0 / 0	Interim analyses	2 / 2	0 / 0	2 / 1	1 / 0
4 / 3	2 / 2	0 / 0	3 / 3	Power	0 / 0	0 / 0	2 / 1	4 / 2
2 / 2	1 / 1	1 / 1	1 / 1	Multiplicity/Type I error	0 / 0	0 / 0	0 / 0	2 / 1

FAL - Final advice letter; Tx - Treatment
